# A Randomized Controlled Trial to Assess the Efficacy of a Pre-operative Virtual Operation Theatre Tour on Anxiety and Patient Satisfaction in Adults Undergoing Elective Surgery

**DOI:** 10.7759/cureus.32337

**Published:** 2022-12-09

**Authors:** Vineet Kumar, Pritam Yadav, Nidhi Bangarwa, Deepika Budhwar, Prashant Kumar, Vandna Arora

**Affiliations:** 1 Department of Anaesthesiology and Critical Care, Pandit Bhagwat Dayal Sharma Post Graduate Institute of Medical Sciences, Rohtak, IND

**Keywords:** surgery, fear, technology, unfamiliar environment, multimedia, audiovisual, spinal anaesthesia, operation theatre environment, audiovisual virtual tour, anxiety

## Abstract

Background: The study objective involves the evaluation of preoperative audiovisual information on the operation theater environment as a tool to relieve anxiety in patients posted for infra umbilical elective surgery under spinal anesthesia. Perioperative anxiety is detrimental to both intraoperative hemodynamic instability and postoperative recovery.

Material and methods:* *The design was a single-center, prospective, randomized control trial. There were 120 patients in this study within the 18-60-year-old age range who were American Society of Anaesthesiologists (ASA) class I-II admitted for infra-umbilical surgery excluding lower segment cesarean section (LSCS) under spinal anesthesia. Patients were randomized into two groups: those who were not exposed to an audiovisual tour (NA) (n = 60) and those who were exposed to an audiovisual tour (A) (n = 60). The measurements are based on the demographic details of the patient; the visual analogue score (VAS); and the Amsterdam Preoperative Anxiety and Information Scale (APAIS) for anxiety, hemodynamic parameters i.e., blood pressure (BP), heart rate (HR), respiratory rate (RR), and patient satisfaction score (PSS) on a five-point Likert scale were noted perioperatively for both the groups.

Results: The main results showed a significant (p-value <0.05) decrease in VAS and APAIS for anxiety, HR, and PSS. This was observed during the intraoperative and postoperative periods in the A group in comparison to the NA group.

Conclusion: The preoperative audiovisual virtual tour of the operation theater effectively reduces perioperative anxiety and stabilizes HR; it also improves the satisfaction of patients undergoing elective surgery under spinal anesthesia.

## Introduction

Anxiety is a feeling of unease, fear, and apprehension in response to any external or internal stimuli that leads to behavioral, emotional, cognitive, or physical symptoms [1]. Preoperative anxiety is usually observed in patients that present for surgery. With the advancements in technology and pharmacology, there has been an expansion of day-care surgeries and surgeries performed under local anesthesia where a patient remains conscious during the procedure, which may induce anxiety [2]. Perioperative anxiety is detrimental as it may cause intraoperative hemodynamic instability and may lead to pain, nausea, vomiting, tachycardia, hypertension, increased risk of infection, delayed recovery, and an increased requirement for anesthesia [1,3,4]. When a patient enters an unfamiliar environment such as an operation theater, the sounds of the monitor alarms, the narrowness of the operation table, low temperature, etc., all these factors enhance anxiety [3,5]. The degree of manifestation depends on various factors, one of which is a patient’s susceptibility to preoperative anxiety as well as age, gender, education status, past experiences, etc. [1,6].

The provision of information to patients plays a significant role in decreasing their anxiety. Patient experiences can be improved by providing them explanations and details of the environment and procedures, highlighting the theater environment. This gives the patient an idea of the function and appearance of the operation theater, making it less unfamiliar and giving the patient an opportunity to ask questions [7,8]. The two most common stressors that cause preoperative anxiety are (1) lack of knowledge and (2) fear of the unknown. Education is the most effective method to reduce anxiety [9]. Entering an unfamiliar and closed space with a lot of machines, monitors, and personnel usually makes an individual anxious. These factors play an important and additive role in increasing the patients’ anxiety other than the procedure itself. With technological advancements, creative methods have been developed to decrease anxiety in surgical patients. For instance, audiovisual multimedia technology is an effective tool to provide patients with some idea about the operation theater environment. It is an easy, time-saving method and has good retention capacity. Education via video gets easily incorporated into the patients’ minds and helps alleviate their fear of the unknown. The preparation and information strategies for the patient in the perioperative period improve the outcome of surgical interventions, shorten hospital stays, and increase patient satisfaction levels [10].

One of the major goals in the practice of administration of anesthesia is reducing the level of the patient’s anxiety. A preoperative assessment provides this unique opportunity to reduce the patient’s anxiety by building a fine rapport and by providing information regarding the environment of the operation theater, the anesthesia administration, and the surgery procedure. This present study was planned to assess the effects of a preoperative audiovisual virtual tour of the operation theater in decreasing anxiety in patients who are undergoing surgeries with spinal anesthesia.

## Materials and methods

Study design

The present prospective, single-blind randomized control trial was conducted in the Department of Anesthesiology and Critical Care in a tertiary hospital of a teaching institute after obtaining approval from the Biomedical Research Ethics Committee, Pandit Bhagwat Dayal Sharma Post Graduate Institute of Medical Sciences, Rohtak with approval number (EC/NEW/INST/2020/874)(No. BREC/22/013) and CTRI No. CTRI/2022/02/040482 under clinical trial registry, India. Witnessed, informed consent was taken from patients.

Patients, endpoints, and assessment

There were 120 patients in the age group of 18-60 years old who were enrolled in the study; they were American Society of Anaesthesiologists (ASA) class I-II admitted for infra-umbilical (excluding lower segment cesarean section {LSCS}) surgery with spinal anesthesia. Patients who had a prior visit to the operation theater, those who are visual and hearing impaired, those with a history of psychiatric/neurological disorder, those with a history of head injury, those with a history of drug abuse, those with a history of alcohol abuse or any psychological trauma in past six months were excluded from the study. The patients who were scheduled for elective surgery under spinal anesthesia were randomly divided into two groups by permuted block randomization: Group I was composed of patients who were not exposed to the audiovisual tour (NA) (n = 60). Group II was composed of patients exposed to the audiovisual tour (A) (n = 60). Demographic details of the patient (age and sex), blood pressure (BP), heart rate (HR), and respiratory rate (RR) were noted preoperatively. All patients were asked to fill out the six-item questionnaire from APAIS for the assessment of their baseline anxiety: 1. I am worried about the anesthesia, 2. The anesthesia is on my mind continuously, 3. I would like to know as much as possible about the anesthesia, 4. I am worried about the procedure, 5. The procedure is on my mind continuously, 6. I would like to know as much as possible [11]. The measurement of agreement with these statements was graded on a five-point Likert scale from 1 (“Not at all”) to 5 (“Extremely"). The group allocations were sealed in serially numbered opaque envelopes prepared by the research coordinator who was not involved in the study.

Group NA were patients who underwent routine bedside pre-anesthetic consultation the day before the surgery. On the other hand, Group A included the patients who, along with the routine bedside pre-anesthetic consultation, were shown a five minutes video clip containing a virtual tour of the preoperative room, the operation theater, and the recovery room using a tablet with a 10-inch screen size during the pre-anesthetic checkup visit on the day before the surgery. A video clip was accompanied by auditory information related to the operation theater environment like monitors, lights, tables, and staff. A prerecorded video clip was validated and standardized by five anesthesiologists with more than five years of experience in anesthesia administration. On the day of the surgery, APAIS for anxiety was noted before the patients were given anesthesia. The HR, BP, and RR were noted at the start of the anesthesia procedure and repeated every five minutes for 20 minutes and then every 10 minutes until the end of the intraoperative surgery for both of the groups. Postoperatively, 10 mins after shifting from the operation theatre (OT) for both groups, the patients were asked to give an estimate of their anxiety based on the visual analogue score (VAS) and to rate their satisfaction on a scale of 1-5 Likert scale.

Statistical analysis

All statistical analyses were performed by using SPSS 22.0 software package (IBM Corp., Armonk, NY). All data were summarized as mean ± SD for continuous variables, numbers, and percentages for categorical variables. The variables were assessed for normality using the Kolmogorov-Smirnov test. A p < 0.05 was accepted as statistically significant.

## Results

Both the groups were matched for their age; gender; and baseline HR, MAP( Mean Arterial Pressure), RR, VAS, and APAIS for anxiety. The difference observed for these baseline characteristics among all four groups was statistically insignificant (Table 1).

**Table 1:** Patient demographics, baseline VAS, HR, MAP, RR, and APAIS HR: heart rate, MAP: mean arterial pressure, RR: respiratory rate, VAS: visual analogue scale, APAIS: Amsterdam Preoperative Anxiety and Information Scale, M: Male, F: Female

	Group NA	Group A	P value
Age (mean± SD)	39.00± 12.96	38.43± 13.06	0.81
Gender (%)	M-46.7; F-53.3	M-51.7; F-48.3	0.58
HR (mean± SD)	76.70± 9.01	76.97± 9.04	0.87
MAP (mean± SD)	92.65± 8.42	92.31± 8.31	0.82
RR	12.17± 0.867	12.25± 0.600	0.54
VAS	64.10± 4.233	64.52± 3.539	0.56
APAIS	12.07± 1.517	11.90± 1.684	0.57

VAS and APAIS: The non-audiovisual group and the audiovisual group showed a significant difference in anxiety scores measured with VAS and APAIS as recorded in the preoperative room (timepoint 0) after being shown the prerecorded video of the operation theater with a p-value of 0.001 for both with the mean value of VAS as 30.25 for the NA group and 24.92 for the A group. The mean APAIS at timepoint 0 was 9.65 for group NA and 7.7 for group A (Table 2).

**Table 2:** Preoperative APAIS (APAIS 0), preoperative VAS (VAS 0), and postop VAS (VAS post) (data expressed as mean ±SD). VAS: visual analogue scale, APAIS: Amsterdam Preoperative Anxiety

	Group NA	Group A	P value
APAIS 0	9.65± 0.88	7.70± 0.67	0.001
VAS O	50.10± 2.95	36.37± 1.90	0.001
VAS Post	30.25± 2.92	24.92± 1.61	0.001

**Figure 1:**
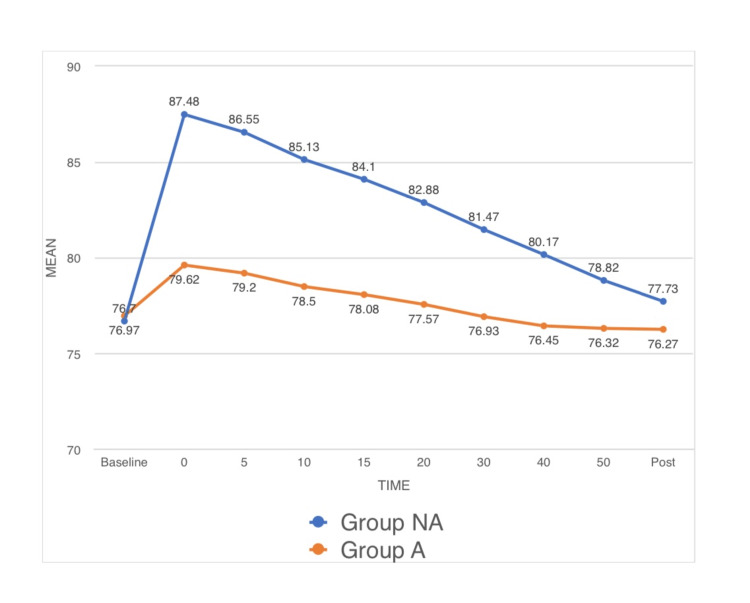
A line diagram showing the preoperative mean HR, the mean HR at time points of 5, 10, 15, 20, 30, and 40 min after the initiation of surgery and the mean HR in the postoperative period. HR: heart rate, Pre: preoperative, min: minute, Post: postoperative.

**Figure 2:**
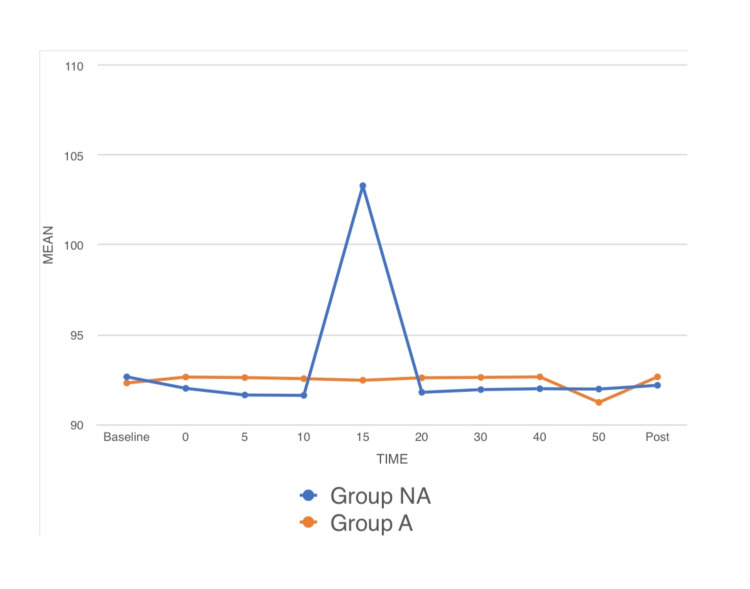
A line diagram showing the preoperative mean MAP, the mean MAP at time points of 5, 10, 15, 20, 30, and 40 min after the initiation of surgery and the mean MAP in the postoperative period. MAP: mean arterial pressure, Pre: preoperative, min: minute, Post: postoperative.

**Figure 3:**
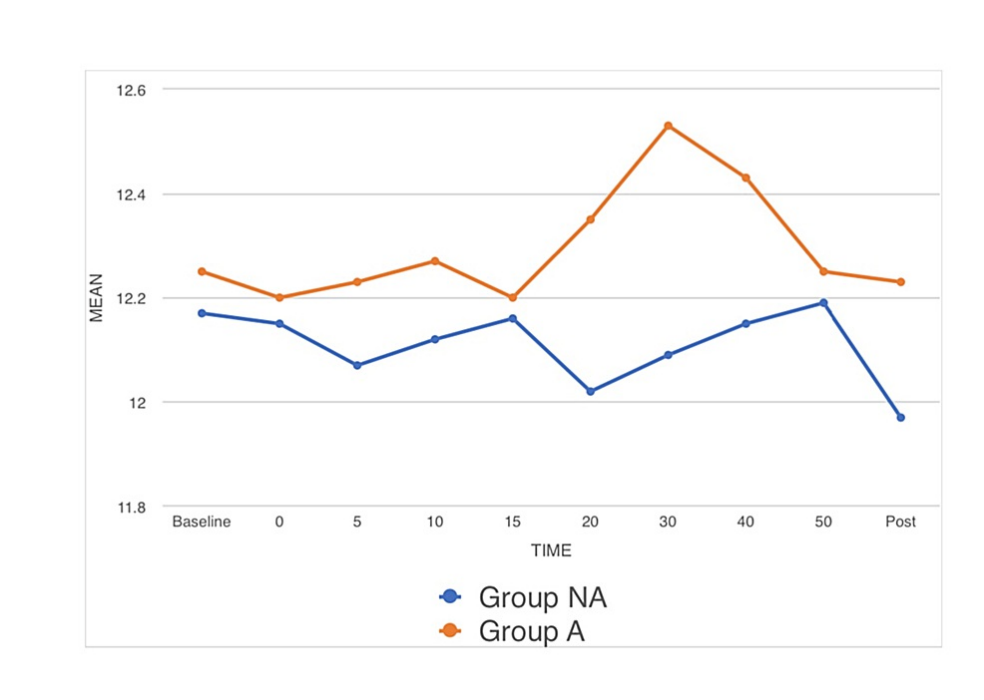
A line diagram showing the preoperative mean RR, the mean RR at time points of 5, 10, 15, 20, 30, and 40 min after the initiation of surgery and the mean RR in the postoperative period. RR: respiratory rate, Pre: preoperative, min: minute, Post: postoperative.

Hemodynamic parameters: Significant difference was observed in HR between both groups (p-value < 0.05) as observed in the preoperative room (time point 0) and at time points 5, 10, 15, 20, 30, and 40 mins post spinal anesthesia. The mean HR for group NA at time points 0, 5, 10, 15, 20, 30, and 40 mins were 87.48, 86.55, 85.13, 84.10, 82.88, 81.47, and 80.17, respectively. The mean HR for group A at time points 0, 5, 10, 15, 20, 30, and 40 mins were 79.62, 79.20, 78.50, 78.08, 77.57, 76.93, and 76.45, respectively (Figure 1). No significant difference was observed in MAP among both groups (p-value > 0.05) as observed in the preoperative room (time point 0) and at time points 5, 10, 15, 20, 30, and 40 mins post spinal anesthesia (Figure 2). No significant difference was observed in RR among both groups (p-value < 0.05) as observed in the preoperative room (time point 0) and at time points 5, 10, 15, 20, 30, and 40 mins post spinal anesthesia (Figure 3).

Patient satisfaction score: Significant difference was noted between both groups with the mean post PSS of 2.47 for group NA and 3.90 for group A with a p-value of 0.001 (Table 3).

**Table 3:** Postoperative PSS (data expressed as mean ± SD). PSS: Patient satisfaction score

	Mean	Standard Deviation	P value
Group NA	2.47	0.72	0.001
Group A	3.90	0.68	0.001

## Discussion

Perioperative anxiety is very common in surgical patients posted for surgery, especially those under regional anesthesia. Perioperative anxiety adds to both the patient's physical and mental stress, leading to delayed surgical healing, physiological challenges, and a negative experience of anesthesia. The unfamiliarity with the operation theater environment and the lack of knowledge seem to be significant contributing factors to patients’ anxiety. Taking advantage of the advancements in modern technology, audiovisual multimedia with a prerecorded video of the operation theater can be shown to patients to make them aware of their surroundings, easing their anxiety. It’s an easy and noninvasive way to calm patients down and tone down their anxiety.

The present study assessed the efficacy of showing a prerecorded audiovisual clip of the operation theater in reducing perioperative anxiety. In this study, patients in both groups were comparable in age, gender, baseline anxiety score, heart rate, mean arterial pressure, and respiratory rate. A significant decrease in VAS, APAIS for anxiety, heart rate, and PSS was observed during intraoperative and postoperative periods in the audiovisual group in comparison to the control group. A study conducted by Helms evaluated the efficacy of video education in improving preoperative anxiety in a bariatric surgical patient. The results were coherent with our study with a significant reduction in anxiety levels using VAS [9]. Similar results were observed in the study by Yesilyurt et al. exploring the effects of preoperative video information on anxiety and satisfaction in patients who are undergoing abdominal surgery [10]. After the patients in the experiment group watched the video information, the anxiety specific to the surgery questionnaire mean scores of the experimental group were lower than before the information (p < 0.001). Satisfaction with nursing care scale mean scores were also higher than those of the control group (p < 0.001) [10]. Our results were coherent with the study conducted by Jlala et al., where patients in the control group experienced an increase in their state of anxiety immediately before surgery (p < 0.001); the patients in the film group, on the other hand, were less anxious before their operation in comparison to those in the control group (p = 0.04). After the operation, there was a decrease in their state of anxiety from the baseline in both groups, but patients in the film group were less anxious than those in the control group (p = 0.005) [12]. A study conducted by Dias et al. showed a significant increase in state anxiety scores before the administration of subarachnoid block in the nonvideo group (p < 0.001). Patients in the video group, however, had significantly lower HR and MAP preoperatively (p < 0.001) [13]. Lin et al. observed similar results of the lower state of anxiety scores in patients after viewing an anesthetic patient information video, and the overall satisfaction was significantly higher in the experimental group than in the control group (p < 0.00) [14]. Moreover, Rajput et al. conducted a study to assess the efficacy of preoperative multimedia-based video information on perioperative anxiety and hemodynamic stability in patients who are undergoing surgery with spinal anesthesia. Patients in the video group showed better/lower anxiety levels than the nonvideo group. Similarly, the hemodynamic parameters were better controlled and showed lesser deviation from the baseline values in the test group in comparison to the control group and showed significant statistical difference (p < 0.001) just before the surgery [15]. Yuzkat et al. enrolled 90 patients to study the effects of showing the operating room on preoperative anxiety and hemodynamics among patients with hypertension. State-Trait Anxiety Inventory scores that were measured on the day of the surgery were lower for the interventional group than those in the noninterventional group (p = .001). Systolic (p = .001, p = .006, respectively); diastolic (p = .001, p = .004, respectively); and heart rate (p = .018, p = .031, respectively) values in the operation room and the preoperative unit were lower in the interventional group than in the noninterventional group. In fact, the number of postponed operations was also lower, recording better patient satisfaction scores in the interventional group [16]. Doering et al. showed a videotape of a patient undergoing a total hip replacement surgery that covered the time period from hospital admission to discharge, strictly keeping the patient’s perspective in relation to the enrolled patients. In comparison with the control group, the preparation group showed significantly less anxiety in the morning before their surgery and in the mornings of the first two postoperative days, and significantly fewer of them had an intraoperative systolic blood pressure increase of more than 15%. The prepared patients also needed less analgesic medication after surgery and had significantly lower cortisol excretion during the preoperative night and the first two postoperative nights [17].

Thus, as observed in our above-mentioned studies, it is evident that patients experience the highest level of anxiety at the induction of anesthetics. The impact of the operating theater environments on patients’ anxiety is in less degree influenced by the sight and hearing of the technical equipment and the surroundings. Being continuously informed and being given the opportunity to ask questions reduce patients’ anxiety [18]. It is important to define the sources of patients' anxieties and show maximum effort for relief because unresolved anxieties in this period may have adverse effects on postoperative healing and may prolong the postoperative hospitalization of the patients [19].

A reduction in the subjective perception of anxiety was not reflected in the objective vital parameters of the patients (MAP, RR) except for the stabilization of HR. Other factors like better interaction through audiovisual activity and psychological support could have contributed to the reduction in anxiety, along with their familiarization with the environment. More studies with large sample sizes and more specific patient populations are required to exactly establish the relationship between environment familiarization and perioperative anxiety.

The limitations of our study include being single-centric, having a small sample size, the nonuniformity in nature of surgeries, and the nonassessment of the subjective anxiety scores intraoperatively. More elaborate subjective scores for anxiety and biochemical markers (e.g., cortisol levels, epinephrine, epinephrine) can be used to better correlate variations in anxiety levels.

## Conclusions

From the observations gathered, it is evident that showing patients a prerecorded audiovisual clip of the operation theater effectively reduces perioperative anxiety, stabilizes HR, and improves the satisfaction score of patients who undergo elective surgery under regional anesthesia. This method is simple, easy to practice, has low cost, and can increase overall subjective patient satisfaction, potentially improving patient safety and clinical outcomes. 
